# Trajectory data of antero- and retrograde movement of mitochondria in living zebrafish larvae

**DOI:** 10.1016/j.dib.2020.105280

**Published:** 2020-02-13

**Authors:** Frank Mieskes, Fabian Wehnekamp, Gabriela Plucińska, Rachel Thong, Thomas Misgeld, Don C. Lamb

**Affiliations:** a Department of Chemistry, Center for Nano Science (CENS), Center for Integrated Protein Science (CIPSM), Nanosystems Initiative Muünchen (NIM), Ludwig Maximilians-Universität München, Munich, Germany; b Munich Cluster for Systems Neurology (SNergy), Center for Integrated Protein Science (CIPSM), German Center for Neurodegenerative Diseases (DZNE), Institute of Neuronal Cell Biology, Technische Universitätt München, Munich, Germany

**Keywords:** Single particle tracking, Orbital tracking, Mitochondria trafficking, Fluorescence, Transport

## Abstract

Recently, a large number of single particle tracking (SPT) approaches have been developed. Generally, SPT techniques can be split into two groups: *ex post facto* approaches where trajectory extraction is carried out after data acquisition and *feedback based* approaches that perform particle tracking in real time [1]. One feedback approach is 3D Orbital Tracking, where the laser excitation beam is rotated in a circle about the object, generating a so called orbit [2,3]. By calculating the particle position from the detected intensity after every orbit in relation to its center, this method allows the microscope to follow a single object in real time. The high spatiotemporal resolution of this method and the potential to optically manipulate the followed object during the measurement promises to yield new deep insights into biological systems [4–7]. By upgrading this approach in a way that the specimen is recentered by a xy-stage on the center of the microscope, particle tracking with this long-range tracking feature is no longer limited to the covered field-of-view. This allows for the observation of mitochondrial trafficking in living zebrafish embryos over long distances. Here, we provide the raw data for antero- and retrograde movement of mitochondria labelled with photo-activatable green fluorescent protein (mitoPAGFP). It relates to the scientific article “Nanoresolution real-time 3D orbital tracking for studying mitochondrial trafficking in vertebrate axons in vivo” [8]. By applying a correlation analysis on the trajectories, it is possible to distinguish between active transport and pausing events with less biasing compared to the mean squared displacement approach.

**Table:** Specifications Table

Subject	Biochemistry, Genetics and Molecular Biology, Biophysics, Neuroscience
Specific subject area	Fluorescence Microscopy, Single Particle Tracking
Type of data	Table Figure
How data were acquired	Hardware: inhouse built confocal microscope based on a Zeiss Axiovert 200 M. For details, see Refs. [8,9] Software: inhouse developed real-time tracking software (LabVIEW)
Data format	Raw Analyzed
Parameters for data collection	Zebrafish larvae (mutant zebrafish line Roy) were embedded in low melting agarose gel. Labelling was carried out by injecting desired UAS construct into eggs immediately after fertilization.
Description of data collection	Data was collected at three days post fertilization at 25 °C in low melting agarose gel. The mitochondrion of interest was photoactivated with 405 nm laser excitation and afterwards tracked using 488 nm excitation. The orbit time was set to 5 ms followed by one 5 ms dark orbit where the specimen was not illuminated. The long-range tracking threshold was set to a threshold of 0.5882 V or 10.18 μm.
Data source location	Department of Chemistry, Ludwig-Maximilians-Universität München, Munich, Germany
Data accessibility	Repository name: Zenodo Data identification number: 10.5281/zenodo.2815430 Direct URL to data: https://zenodo.org/record/2815430#.Xfnk-PwxmUk Analysis Program: Repository name: Gitlab Direct URL: https://gitlab.com/frmie/Orbital-Tracking-Zebrafish2019
Related research article	F.Wehnekamp, G. Plucińska, R. Thong, T. Misgeld, D. C. Lamb, Nanoresolution real-time 3D orbital tracking for studying mitochondrial trafficking in vertebrate axons in vivo, eLIFE, https://doi.org/10.7554/eLife.46059.001 [8].

**Table:** 

**Value of the Data** • The data provide long traces (up to 111,538 data points and displacements of up to 100 μm) with high spatiotemporal resolution including stationary and directed motion of different velocities. • The data can be used for developing more detailed models of mitochondrial transport and looking at the transition mechanisms between different motional behaviors. • Research on neurological diseases may benefit from a detailed analysis of mitochondrial transport as the transport speed and transition probabilities may be affected.

## 1 Data description

Single particle tracking has become a powerful technique for investigating the dynamics of biomolecules and complexes [1]. Here, we focus on data collected using three-dimensional orbital tracking [2,3], which provides a high temporal and spatial resolution and has already yielded new biological insights (see e.g. [[4], [5], [6], [7]]. In this feedback based approach, the trajectory of the particle is written to disk during the measurement. Trajectories of individual mitochonria being transported in the axon of sensory neurons in zebra fish embryos are the data we provide in this article (see [8] for details). The trajectories contains a wealth of information regarding the behavior of the particles that can extracted using various analysis methods. The dataset represents the raw data of several tracked mitochondria in the antero- and retrograde directions. Each .txt file includes all information with respect to one tracked mitochondrion. The file header contains information about the experimental settings, i.e. defined orbit time and -radius, number of delay orbits in which the sample was not illuminated with light and long-range tracking events where the sample is recentered on the microscope (0: disabled; 1: activated) with the corresponding border at which the repositioning is performed. The information available in the .txt files is listed in Table 1.

**Table 1:** Overview of raw date file including file header and data entries.

File Header	
Entry	Description
File Path	Original file path of raw data
Date	Date when experiment was carried out
Time	Begin of experiment
Orbit Time [ms]	User defined time of orbit rotation
Orbit Radius [V]	User defined size of orbit
Tracking Threshold [Hz]	Threshold for distinguishing between the execution of tracking or search algorithm
Delay Orbits	Number of dark orbits
Number of Particles	Number of tracking channels in the experiment
Long Range	Information regarding activation of long range tracking mode (0: disabled; 1: activated)
Long Range Threshold [V]	User defined threshold at which the repositioning of stage is to be executed during a long-range tracking experiment
Data Entries	

Column	Description of entry
1 – 3	Position information (x,y,z)
4	Orbit number
5	Calculated orbit time (including delay orbits and long-range tracking events)
6 & 7	Total detected signal of each detector during the given orbit. Two detectors are used to provide the z-position given in column 3
8	Camera frame for the wide-field detection
9	Tracking (0: inactive; 1: active)
10 & 11	When long-range tracking is enabled, this provides information on whether the sample is being tracked or the microscope stage is being repositioned in x (column 10) and y (column 11) (0: repositioning is inactive; 1: sample is being repositioned)

The first three columns from the data entries provide the particle position information for each coordinate axis. The distances have been updated for any long-range tracking events. In the fourth and fifth columns, the orbit number as well as the orbit time for each orbit during the measurement are saved. For experiments where the particle of interest is moving slowly in comparison to the maximum tracking speed of the setup, dark orbits can be introduced where the excitation laser is turned off during the orbit. This allows the particle to be tracked over longer times with less photobleaching, but at a reduced temporal resolution. Dark orbits are not included in the data file as they do not contribute any new tracking information (but are given an orbit number, which will be missing in the file) and the timing of the dark orbit is included in the determination of the calculated orbit time. In these experiments, data were collected at 5 ms per orbit with every second orbit being a dark orbit, leading to an overall temporal resolution of 10 ms. The detected intensity of each detector per orbit is listed in columns six and seven. When a simultaneous wide field image is measured, the camera frame number is given in column eight. In column nine, information is given of whether a particle is being actively tracked in this orbit. For example, in the beginning of the experiment, when the instrument is looking for a particle, this will be zero. Also, when the intensity of the particle drops below a given threshold indicating photobleaching or that a particle has left the orbit, this will be zero. In the last two columns, information regarding the status of long-range tracking is given. When long-range tracking is enabled, particles that reach a predefined distance from the center of the field of view are recentered on the microscope. The orbital tracking is performed by adding an offset to the orbiting galvanometer mirrors, which allows fast feedback of the system. Hence, the laser tracks the particle. The quality of tracking decreases as the particle moves farther from the center of the field of view where everything is optimally aligned. Hence, upon reaching a predetermined threshold, the microscope stage is repositioned to place the particle at the center of the field-of-view and the galvanometer mirrors are also recentered. This takes 30–70 ms, which is typically much longer than the time of a single orbit. Hence, these columns indicate whether a reposition event in x (column 10) or y (column 11) is taking placed during the recorded orbit.

All position values including the particle position (columns 1–3), orbit radius (file header) and long range threshold (file header) are stored as voltages in the corresponding hardware. To transform this information into the trajectory of the particle, the particle position needs to be multiplied by the determined scaling factors (lateral: 17.30 μm/V; axial: 10.00 μm/V).

## 2 Experimental design, materials, and methods

### 2.1 Sample preparation and data acquisition

A mutated zebrafish line (Roy) was used in these experiments [[10], [11], [12], [13]]. Labelling with mitoPAGFP was carried out by injecting UAS constructs into fertilized eggs [14]. For tracking measurements on the microscope, animals were embedded in low melting agarose gel. The temperature was set to 25 °C during the experiments. The mitoPAGFP of single mitochondria was activated by scanning a region in the vicinity of the particle with 405 nm light and then tracked with 488 nm excitation. As we were tracking moving mitochondria, the mitochondria quickly moved away from the photoactivated area and we did not have problems with multiple photoconverted mitochondria interfering with the tracking algorithm. During tracking experiments with enabled long range tracking, the specimen was automatically recentered in the field of view by a xy-stage when the mitochondrion was crossing a pre-defined position threshold of 0.5882 V or 10.18 μm. Detailed information regarding sample preparation and data acquisition is provided in the related paper [8].

### 2.2 Correlation analysis

**Fig. 1:**
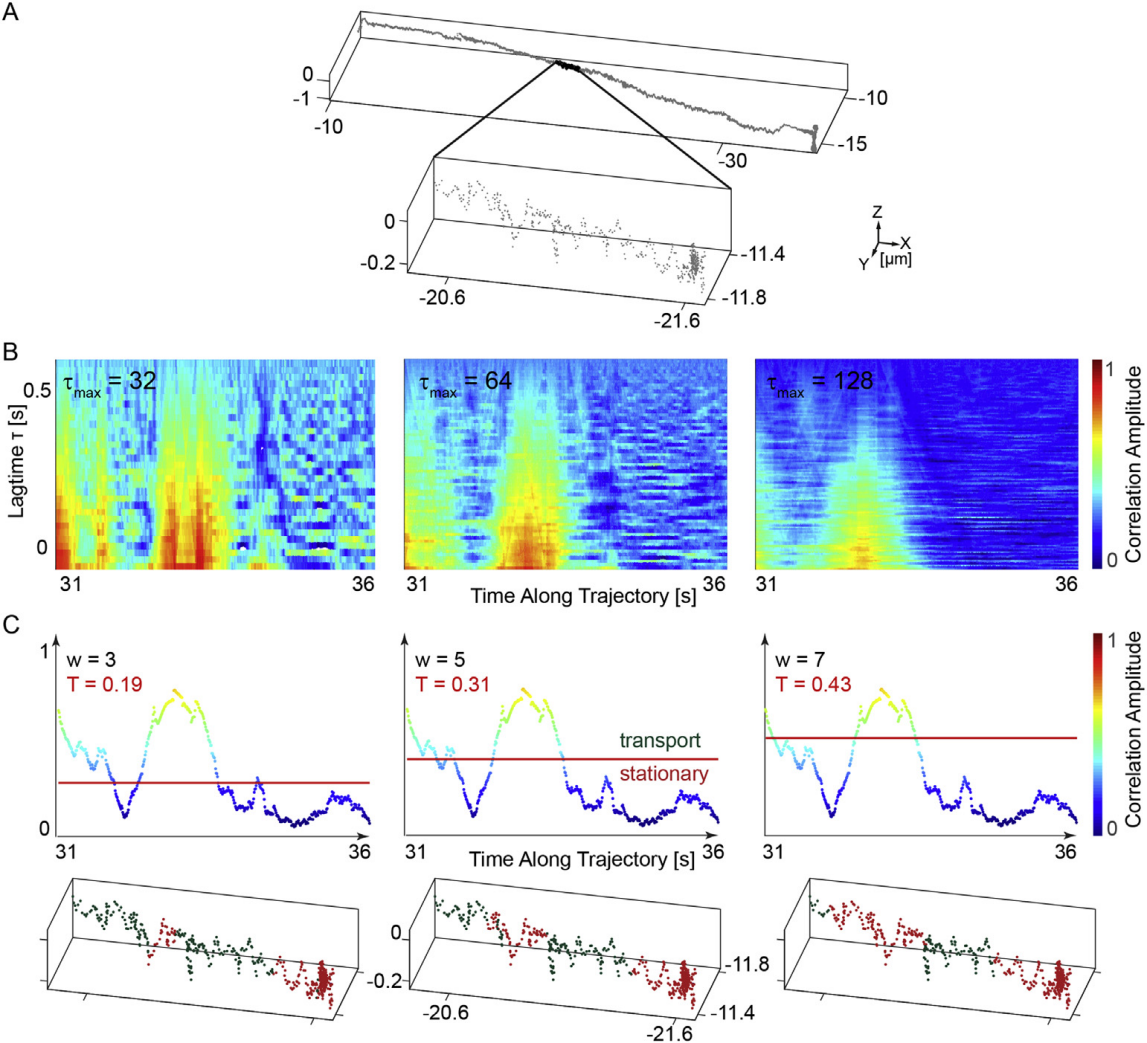
Correlation analysis of a mitochondrial retrograde trajectory. (a) An example trajectory and zoom in of a moving mitochondrion in the retrograde direction with a time resolution of 100 Hz. (b) Correlation carpets of the lateral angles Φ(t) between consecutive orbits with different sliding windows $\tau_{\text{max}}$. (c) The correlation amplitude determined from the sum of the correlation function over a sliding window of 64 data points is plotted. Three different weighting factors corresponding to thresholds of 3, 5, and 7 times the standard deviation of the correlation function calculated from the randomized trace (Equation (2)) are shown in red. The lower plots show the influence of the different thresholds on the separation of regions of directed transport (shown in green) and stationary phases (shown in red) for the zoomed in region of the trace in panel (a).

To distinguish between active transport and stationary states, we performed a correlation analysis on the angle between consecutive positions. The idea behind the analysis is that stationary states will have a random distribution of angles where as directed transport will have a preferred direction of motion. If the reader wishes to compare their analysis of the trajectories with what we published (Figure 2in Ref. [8]), we provide a short description of how we analyzed the data. The axis for the correlation was determined by a line connecting the position between the beginning and end of the trajectory in the *x*, *y* plane. The displacement in *z* was small in comparison to the lateral displacement for the trajectories and was ignored. The lateral angle between two orbits Φ(t) was calculated along the trajectory. The correlation of the lateral angle was then calculated along the trajectory:

(1) $$Cor\left(t,\tau \right)=\frac{1}{\left(n-\tau \right)}\sum_{i=t}^{t+\tau_{max}-\tau }\text{Φ}\left(i\right)\text{Φ}\left(i+\tau \right)$$

where τ is the correlation lag time, *t* the time along the orbit, *n* the number of data points and $\tau_{\text{max}}$ the size of the correlation window. An example of the correlation analysis is shown in Fig. 1 for a retrograde mitochondrial trajectory obtained at 100 Hz (Fig. 1a). The correlation carpets for the zoomed in region of the trajectory were calculated according to equation (1) for three different correlation windows $\tau_{\text{max}}$ = 32, 64 and 128 data points (Fig. 1b). As shown, the noise as well as the sensitivity are strongly dependent on the size of $\tau_{\text{max}}$. For our analysis, we choose a time window of $\tau_{\text{max}}$ = 64 data points. For determining the threshold between active transport and stationary states for each trajectory, the angles were randomized and the correlation analysis was repeated for the randomized lateral angles *Φ*_*rand*_ obtaining a *Cor*_*rand*_. As the distribution of angles in a trajectory with a large total displacement already contains a bias, we added a negative copy of the trajectory to the data before performing the randomization. The threshold *T* was set by looking for a correlation amplitude that deviated by more than five standard deviations (the weighting factor *w* below) from the average of the randomized correlation function, calculated with following equation

(2) $$T=M\left(Cor_{rand}\right)+w*\sigma \left(Cor_{rand}\right)$$

where *M* is the mean of *Cor*_*rand*_ and *σ* the corresponding standard deviation. Fig. 1c illustrates the influence of three different weighting factors *w* to determine the threshold *T*. In the lower plot, the zoomed in region of the trajectory in Fig. 1a is separated into stationary phases (shown in red) and transport (shown in green) respectively. As described in the main paper, the weighting factor *w* was set to five [8]. Each region of the trajectory above the threshold was treated as a single, directed transport event in which the distance, duration and velocity could be determined. The code for this correlation analysis was written in Matlab and is available online: https://gitlab.com/frmie/Orbital-Tracking-Zebrafish2019. It is also worth mentioning the software package of Christoph Gohlke [15], who has written a Python library to analyze data generated by SimFCS [16]. To analyze our raw data with Chirstoph Gohlke's software, it is currently necessary to write a read-in function.
